# Multicenter, randomized, double-blind, placebo-controlled phase 3 study of mogamulizumab with open-label extension study in a minimum number of patients with human T-cell leukemia virus type-1-associated myelopathy

**DOI:** 10.1007/s00415-024-12239-x

**Published:** 2024-03-02

**Authors:** Tomoo Sato, Masahiro Nagai, Osamu Watanabe, Tatsuro Misu, Norihiro Takenouchi, Ryuichi Ohkubo, Satoshi Ishihara, Yoshio Tsuboi, Masahisa Katsuno, Masanori Nakagawa, Takuya Matsushita, Yasuhiro Aso, Eiji Matsuura, Takashi Tokashiki, Akihiro Mukaino, Hiroaki Adachi, Kaoru Nakanishi, Yusuke Yamaguchi, Saaya Yamaguchi, Yoshihisa Yamano

**Affiliations:** 1 Department of Rare Diseases Research, Institute of Medical Science, St. Marianna University School of Medicine, Kawasaki, Japan; 2 Department of Neurology, St. Marianna University School of Medicine, Kawasaki, Japan; 3 Department of Neurology and Clinical Pharmacology, Ehime University Hospital, Toon, Japan; 4 Department of Neurology, Kagoshima City Hospital, Kagoshima, Japan; 5 Department of Neurology, Tohoku University Hospital, Sendai, Japan; 6 Department of Microbiology and Department of Neurology, Kansai Medical University, Hirakata, Japan; 7 Department of Neurology, Fujimoto General Hospital, Miyakonojo, Japan; 8 Department of Cardiovascular Medicine, Nephrology and Neurology, Graduate School of Medicine, University of the Ryukyus, Okinawa, Japan; 9 Department of Neurology, Fukuoka University, Fukuoka, Japan; 10 Department of Neurology, and Department of Clinical Research Education, Nagoya University Graduate School of Medicine, Nagoya, Japan; 11 Department of Neurology, Kyoto Prefectural University of Medicine, Kyoto, Japan; 12 Department of Neurology, Neurological Institute Graduate School of Medicine, Kyushu University, Fukuoka, Japan; 13 Department of Neurology, Oita Prefectural Hospital, Oita, Japan; 14 Department of Neurology and Geriatrics, Kagoshima University Graduate School of Medical and Dental Sciences, Kagoshima, Japan; 15 Division of Neurology, National Hospital Organization Okinawa National Hospital, Ginowan, Japan; 16 Department of Molecular Neurology and Therapeutics, Kumamoto University Hospital, Kumamoto, Japan; 17 Department of Neurology, University of Occupational and Environmental Health School of Medicine, Kitakyushu, Japan; 18 Clinical Development, R&D Division, Kyowa Kirin Co., Ltd., Tokyo, Japan

**Keywords:** KW-0761, Mogamulizumab, Human T-cell leukemia virus type 1-associated myelopathy/tropical spastic paraparesis, Osame motor disability score, Neopterin, CXCL10

## Abstract

Human T-cell leukemia virus type 1 (HTLV-1)-associated myelopathy/tropical spastic paraparesis (HAM/TSP) is a chronic neurodegenerative disease. This multicenter, randomized phase 3 study evaluated the efficacy and safety of 0.3 mg/kg intravenous mogamulizumab, a monoclonal antibody targeting-CC chemokine receptor 4, every 12 weeks in HAM/TSP patients. This study comprised a 24-week double-blind, placebo-controlled period, 24-week open-label period, and extension treatment period. The primary endpoint was the proportion of patients with a ≥ 1-grade improvement in the Osame motor disability score (OMDS). Secondary endpoints were changes in HTLV-1 proviral load, 10-m timed walk, cerebrospinal fluid (CSF) neopterin levels, and safety. The exploratory endpoint was CSF chemokine C-X-C motif ligand 10 (CXCL10) levels. Thirty-four and 33 patients were randomized to mogamulizumab and placebo arms, respectively. At the end of the double-blind period, no significant difference was found in the OMDS improvement rate or other secondary efficacy endpoints assessing motor activities. However, the mogamulizumab arm showed a significant decrease in HTLV-1 proviral load (− 59.39 ± 29.91% vs. placebo 2.32 ± 36.31%) and CSF neopterin (*p* < 0.001)/CXCL10 levels (*p* = 0.004). The baseline OMDS pattern and the 60–80% HTLV-1 proviral load reduction were sustained through the open-label and extension treatment periods. Although a higher incidence of rash (69.2%) was reported, the safety profile was similar compared with a previous phase 1/2a study. We found no significant difference in clinical benefit; however, mogamulizumab may provide long-term clinical benefit by preventing disease progression, as CSF neopterin/CXCL10 levels are associated with long-term prognosis in HAM/TSP.

Clinical Trial Registration Number: NCT03191526 (registered date: 6-June-2017).

**Supplementary Information:**

The online version contains supplementary material available at 10.1007/s00415-024-12239-x.

## Introduction

Human T-cell leukemia virus type 1 (HTLV-1)-associated myelopathy/tropical spastic paraparesis (HAM/TSP) is a chronic neurodegenerative disease characterized by progressive spastic paralysis of the spinal cord and occurs in HTLV-1 carriers [1]. Although most HTLV-1 carriers are asymptomatic, patients with HAM/TSP develop symptoms such as gait disturbance, bladder and bowel dysfunction, weakness, and lower limb paralysis [2–8]. At least 5–10 million people worldwide are infected with HTLV-1; of these, 1.08 million people were reportedly from Japan [9]. Among these carriers, the number of patients with HAM/TSP in Japan is estimated at approximately 3000, and the lifetime incidence of HAM/TSP is approximately 0.25% among HTLV-1 carriers [10]. HAM/TSP is characterized by gradual progression of walking disability in approximately 70–80% of all patients and relatively rapid progression of walking disability in approximately 20% of patients within 2 years after disease onset [11]. A small proportion of patients (< 10%) have relatively mild disease without progression. HAM/TSP is treated mainly using corticosteroids with anti-inflammatory activity and interferon-alpha (IFN-α), with immunomodulatory and antiviral activity [7]. Although these therapeutic agents may be effective short-term, they have little effect on improving the long-term prognosis [12].

Mogamulizumab (KW-0761) is a humanized anti-CC chemokine receptor 4 (CCR4) defucosylated monoclonal antibody that kills CCR4-positive T-cells via antibody-dependent cellular cytotoxicity [2]. Mogamulizumab was approved in Japan in March 2012 for the treatment of relapsed or refractory CCR4-positive adult T-cell leukemia/lymphoma, which is also caused by HTLV-1 [13]. Mogamulizumab was subsequently approved for relapsed or refractory CCR4-positive peripheral T-cell lymphoma, relapsed or refractory cutaneous T-cell lymphoma, and chemotherapy-untreated CCR4-positive adult T-cell leukemia/lymphoma, combined with other antineoplastics. Outside Japan, mogamulizumab was approved for relapsed or refractory mycosis fungoides and Sézary syndrome in adults with a history of systemic treatment [14, 15].

Previous studies demonstrated a high rate of HTLV-1 infection in CCR4-positive T-cells in patients with HAM/TSP and in HTLV-1 carriers [16–18]. Therefore, we performed an investigator-initiated phase 1/2a clinical study of mogamulizumab at St. Marianna University School of Medicine in November 2013 [19], and a long-term safety and efficacy study of mogamulizumab [20]. The results demonstrated that mogamulizumab dose-dependently reduced the peripheral proviral load, reduced the levels of the inflammatory markers chemokine C-X-C motif ligand 10 (CXCL10) and neopterin in cerebrospinal fluid (CSF), and improved clinical symptoms, such as spasticity and motor disability.

The current study was a phase 3 study to verify the efficacy of mogamulizumab in a larger population. The study was also designed to evaluate the long-term efficacy and safety of mogamulizumab with an additional open-label period and extension treatment period in patients with HAM/TSP. We herein report the results of this phase 3 study, which was terminated in May 2021 owing to futility in achieving the study's primary objective.

## Methods

### Study design

**Fig. 1:**
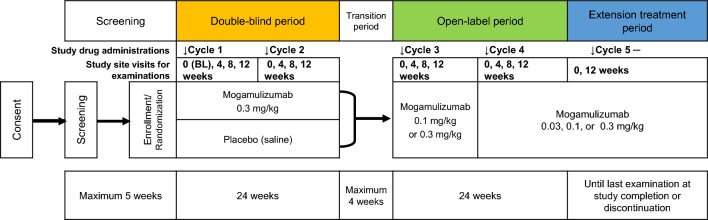
Study design. Vertical arrows indicate timing of either mogamulizumab or placebo administrations. *BL* baseline

This was a multicenter, randomized, double-blind, placebo-controlled phase 3 study with an open-label period and an extension treatment period to evaluate the safety and efficacy of intravenous mogamulizumab at 12-week intervals in patients with HAM/TSP (Fig. 1). The study comprised a ≤ 5-week screening period, a 24-week double-blind period, a ≤ 4-week transition period, a 24-week open-label period, and an extension treatment period that was planned to last until mogamulizumab was approved for the additional indication of HAM/TSP or until study termination, whichever occurred first.

After providing written consent, patients were assessed, enrolled, and randomized in a 1:1 ratio to either the mogamulizumab 0.3-mg/kg arm or placebo arm at least 1 day before the start of the study treatment (Day − 1). Patients began receiving either mogamulizumab or saline as placebo within 5 weeks after the pre-enrollment assessment. One cycle comprised 12 weeks, with the day of study drug administration designated Day 1 (Week 0). During the double-blind period, patients received mogamulizumab at 0.3 mg/kg or placebo twice at 12-week intervals (on Cycle 1–Day 1 and Cycle 2–Day 1). During the transition period, patients received neither mogamulizumab nor placebo under the double-blind condition. During the open-label and extension treatment periods, the 0.3-mg/kg administration was repeated at 12-week intervals for all patients. The dose of mogamulizumab was calculated based on the patient’s body weight on Day 1 of each cycle, and adjusted in accordance with the dosing adjustment criteria.

To prevent or reduce acute infusion reactions and cytokine release syndrome, patients received antihistamines (diphenhydramine, 30–50 mg/dose or d-chlorpheniramine maleate, 2 mg/dose) and antipyretic analgesics (acetaminophen, 300–500 mg/dose) 30–60 min before mogamulizumab infusion.

The following drugs and therapies were prohibited throughout the study period unless the investigators determined that treatment was necessary because of treatment-emergent adverse events (TEAEs): biologics, systemic immunosuppressants, IFN-α, corticosteroid pulse therapy, and drugs and therapies that might affect the efficacy evaluation of mogamulizumab.

Changes in the dosage and initiation of treatment with the following drugs were prohibited until the Cycle 4–Week 12 examination: systemic corticosteroids, salazosulfapyridine, prosultiamine, vitamin C (≥ 1.5 g/day), and pentosan polysulfate. Changes in the dosage and initiation of treatment with the following drugs were prohibited until the Cycle 2–Week 12 examination: tizanidine hydrochloride, eperisone hydrochloride, oral baclofen, propiverine hydrochloride, solifenacin succinate, imidafenacin, prazosin hydrochloride, distigmine bromide, pregabalin, duloxetine hydrochloride, amitriptyline hydrochloride, and clonazepam.

### Dosing adjustment

Patients who met the dosing criteria, determined using the tests performed the day before administration, received mogamulizumab. Dosing was postponed if patients did not meet the dosing criteria. The maximum duration of postponement was 4 weeks in the double-blind period and 8 weeks in the open-label and extension treatment periods for each cycle, and patients were withdrawn from the study if administration could not be resumed within each allowable duration. If patients met all the dosing criteria after postponing the dosing, the study treatment was resumed with mogamulizumab at 0.3 mg/kg or placebo during the double-blind period and with mogamulizumab at the same dose as that used before the dose postponement during the open-label and extension treatment periods. However, a lower dose of 0.1 mg/kg or 0.03 mg/kg with a minimum dose of 0.03 mg/kg was administered if patients met criteria related to TEAEs and laboratory analysis results. A dose increase was allowed if the patients whose dose was postponed during the open-label and/or extension treatment period(s) met all of the dosing criteria after resuming the dosing without another dose postponement.

### Randomization

Eligible patients were randomized (1:1) to either mogamulizumab or placebo using a dynamic allocation strategy stratified by the study site, maintenance corticosteroid therapy (ongoing and none at screening), and the Osame motor disability score (OMDS) (< 5 and ≥ 5) at screening through the registration center.

### Patients

Eligible patients were ≥ 20 years old and had been diagnosed with HAM/TSP in accordance with the diagnostic criteria of the World Health Organization (WHO) [7] with a positive test result for anti-HTLV-1 antibodies in serum and CSF and with exclusion of other diseases by spinal magnetic resonance imaging. Patients were required to have a ≥ 1-year history of HAM/TSP and ongoing medication for HAM/TSP with no changes for 3 months before enrollment or an inadequate response or intolerance to prior medication. Patients on maintenance corticosteroid therapy must have been receiving ≤ 10 mg/day of prednisolone equivalent continuously for at least 3 months before enrollment. Patients must not have experienced a change in the degree of motor dysfunction by OMDS for at least 3 months before the date of screening as judged by the investigators or subinvestigators, and were required to have an OMDS of ≥ 3 and the ability to walk ≥ 10 m at screening (use of a single- or double canes was allowed).

Patients were excluded from the study if they had notable concomitant diseases. Patients with a positive viral infection test result for hepatitis B (HB) and C, and human immunodeficiency virus at screening, were excluded, with the exception of patients who had a negative result for HB surface antigen with a positive result for HB core antigen antibody and/or HB surface antigen antibody, with a subsequently measured HB virus DNA level below the limit of detection (HB virus DNA levels were measured every 4 weeks). The following patients were also excluded: those with a concurrent spinal cord compression lesion (e.g., cervical spine disease, disc herniation, or ossification of the ligamentum flavum), with the exception of conditions that did not affect the efficacy evaluation in the study as judged by the investigator or subinvestigator.

### Endpoints

The primary efficacy endpoint was the improvement rate in the OMDS (score range: 0–13, with higher scores indicating greater disability) (Supplementary Table 1), presented as the number and proportion of patients with a ≥ 1-grade improvement in the OMDS at all three time points in Cycle 2–Weeks 4, 8, and 12 from baseline. A degree of improvement in the OMDS was defined as the smallest change in the OMDS among the three time points in Cycle 2–Week 4, 8, or 12 (with a decrease in the OMDS indicating improved and an increase indicating worsened). The main secondary efficacy endpoints were the mean change in the OMDS from baseline over Cycle 2–Week 4, 8, or 12; mean OMDS; 10-m timed walk test (mean of two 10-m walking times); and the mean percentage change in the HTLV-1 proviral load in peripheral-blood mononuclear cells (PBMCs) at each examination time point. Other secondary efficacy endpoints were muscle spasticity (higher right or left knee score on the modified Ashworth scale [MAS], which assessed knee extensor and flexor muscles using grades 0, 1, 1+, 2, 3, and 4, with higher grades indicating more severe spasticity); overall improvement (Clinical Global Impression-Improvement Scale [CGI-I] and visual analogue scale [VAS] scores); urinary dysfunction (Overactive Bladder Symptom Score [OABSS], range: 0–15, with higher scores indicating more severe urinary urgency; and International Prostate Symptom Score [I-PSS], range: 0–35, with higher scores indicating more difficulty urinating); sensory dysfunction (lower extremity numbness and pain on VAS); and CSF neopterin level (marker of disease activity) at each measurement point. The CSF CXCL10 level was analyzed as an exploratory endpoint. The safety endpoints were TEAEs, laboratory analysis results, vital signs, and standard 12-lead electrocardiogram.

### Statistical analysis

#### Sample size

On the basis of the efficacy rate of the abovementioned phase 1/2a study [19] and the improvement rate in the placebo group of a double-blind randomized controlled study [21] that evaluated the efficacy of IFN-α in patients with HAM/TSP, we determined that 52 patients (26 patients in each arm) were required to detect a difference in the improvement rate between arms using the Wilcoxon rank sum test with 90% power, assuming that the 1- and 2-grade improvements were both 16.7% in the 0.3-mg/kg dosage arm of the phase 1/2a study and 0% in the placebo arm.

#### Statistical methods

The efficacy endpoints were analyzed in the full analysis set, which excluded patients who received no study drug or had no post-dosing OMDS data. Safety was assessed in the safety analysis set, which excluded patients who received no study drug.

All summary data were provided for each treatment arm. Unless otherwise specified, categorical data were presented as frequency and percentage, and continuous data were presented as the number of patients, mean, standard deviation (SD), minimum, median, and maximum. For the efficacy analyses, between-arm differences were assessed using the Wilcoxon rank sum test and t-test, with the corresponding two-sided 95% confidence interval. Statistical analyses were performed using SAS version 9.4 (SAS Institute Japan, Tokyo, Japan). TEAEs were summarized by system organ class and preferred term in accordance with the Medical Dictionary for Regulatory Activities version 24.0. The number and proportion of patients were obtained for all TEAEs and each event. TEAEs that occurred during the transition period were handled as TEAEs that occurred during the double-blind treatment period. Additionally, TEAEs that occurred during the overall study period were summarized for all patients who received at least one dose of mogamulizumab.

This study was performed in accordance with the ethical standards of the Declaration of Helsinki and in compliance with the Ministerial Ordinance on Good Clinical Practice for Drugs (Ordinance of the Ministry of Health and Welfare No. 28, 1997), Pharmaceutical Affairs Law, Ministerial Ordinance on the Partial Revision of the Ordinance, and other related notifications. This study was approved by the internal review board at each study site, and written informed consent was obtained from all participants prior to study initiation.

## Results

### Demographic and clinical characteristics

**Fig. 2:**
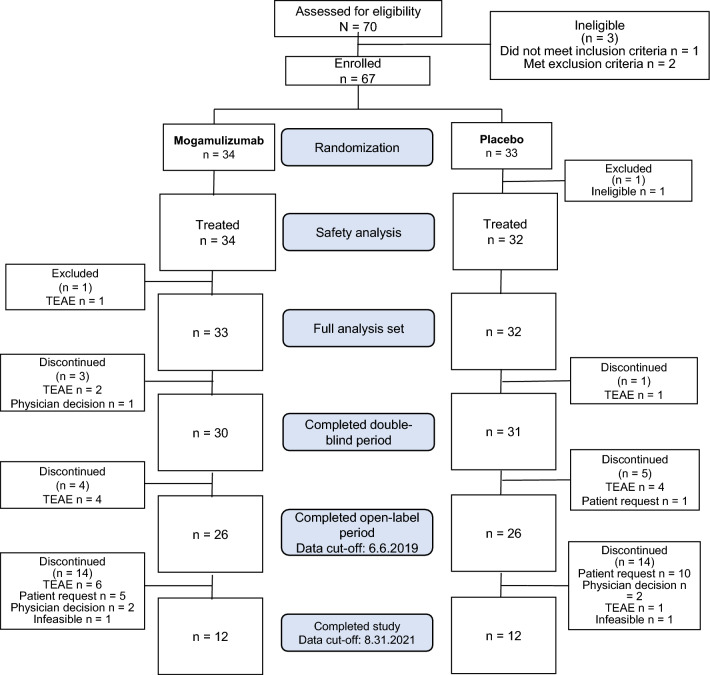
Patient flowchart. *TEAE* treatment-emergent adverse event

Between June 2017 and May 2018, 70 patients provided written informed consent at 16 study sites. Of these, three patients were ineligible (laboratory criteria not met at screening in one patient and significant concomitant diseases in two patients). Therefore, 67 patients were enrolled in the study and randomized to the mogamulizumab arm (34 patients) and placebo arm (33 patients) (Fig. 2). Subsequently, one (1.5%) patient in the placebo arm was excluded because the patient was found ineligible before initiating treatment. Therefore, the remaining 66/67 (98.5%) patients received either mogamulizumab (34 patients, 100%) or placebo (32 patients, 97.0%) (safety analysis set). In the full analysis set, 33 (97.1%) and 32 (97.0%) patients in each arm were included in the efficacy analyses (total: 65 patients), excluding 1 patient in the mogamulizumab arm owing to a TEAE.

As of the data cut-off date of 6 June 2019, 30 patients in the mogamulizumab arm and 31 patients in the placebo arm completed the double-blind period, and 26 patients in each arm completed the double-blind and open-label periods. After the study sponsor made the decision to discontinue the study, and all enrolled patients completed or discontinued the study by 31 August 2021, 12 (35.3%) patients in the mogamulizumab arm and 12 (36.4%) in the placebo arm completed the study (total: 24 [35.8%]), and 22 (64.7%) and 21 (63.6%) patients in each respective arm discontinued the study (total: 43 [64.2%] patients). The main reasons for discontinuation were TEAEs in the mogamulizumab arm (13 patients) and withdrawal by patients in the placebo arm (11 patients).

The mean ± SD patients’ age in the mogamulizumab and placebo arms was 62.2 ± 9.1 years and 60.1 ± 10.0 years, respectively, and the mean ± SD weight was 56.37 ± 10.15 kg and 59.63 ± 11.91 kg, respectively (Table 1). The mogamulizumab arm had a slightly higher proportion of women (72.7%, 24/33 patients) vs. the placebo arm (59.4%, 19/32 patients). The mean ± SD duration of HAM/TSP symptoms in the mogamulizumab arm (12.2 ± 7.9 years) was 2 years shorter vs. the placebo arm (14.3 ± 8.6 years). The other baseline characteristics were well-balanced between the two arms.

**Table 1:** Baseline characteristics of the patients (full analysis set)

Variable		Mogamulizumab *n* = 33	Placebo *n* = 32
Sex	Female	24 (72.7)	19 (59.4)
	Male	9 (27.3)	13 (40.6)
Age (years)	Mean ± SD	62.2 ± 9.1	60.1 ± 10.0
	< 65	20 (60.6)	19 (59.4)
	≥ 65	13 (39.4)	13 (40.6)
Weight (kg)	Mean ± SD	56.37 ± 10.15	59.63 ± 11.91
Height (cm)	Mean ± SD	157.26 ± 7.81	158.63 ± 8.18
BMI (kg/m^2^)	Mean ± SD	22.73 ± 3.14	23.58 ± 3.94
Duration of HAM/TSP symptom (years)	Mean ± SD	12.20 ± 7.95	14.30 ± 8.64
	< 10	14 (42.4)	9 (28.1)
	≥ 10	19 (57.6)	23 (71.9)
Walking aids	Unnecessary	11 (33.3)	10 (31.3)
	Unilateral cane	10 (30.3)	12 (37.5)
	Bilateral cane	12 (36.4)	10 (31.3)
Therapy for HAM/TSP (before screening) (multiple choices)	Corticosteroids	32 (97.0)	25 (78.1)
	IFN-α	7 (21.2)	8 (25.0)
	Salazosulfapyridine	4 (12.1)	1 (3.1)
	Vitamin C (≥ 1.5)g/day)	1 (3.0)	1 (3.1)
Medical history (ongoing at screening)	No	1 (3.0)	2 (6.3)
	Yes	32 (97.0)	30 (93.8)
Medical history (before screening)	No	19 (57.6)	14 (43.8)
	Yes	14 (42.4)	18 (56.3)
Maintenance corticosteroid therapy (ongoing at screening)	No	10 (30.3)	12 (37.5)
	Yes	23 (69.7)	20 (62.5)
Self-catheterization	No	25 (75.8)	25 (78.1)
	Yes	8 (24.2)	7 (21.9)
Baseline OMDS	Mean ± SD	4.7 ± 1.0	4.9 ± 0.9
	≥ 3 to < 5	13 (39.4)	10 (31.3)
	≥ 5 to ≤ 6	20 (60.6)	22 (68.8)

Data are presented as *n* (%) of patients unless otherwise indicated

*BMI* body mass index, *HAM/TSP* human T-cell leukemia virus type 1-associated myelopathy/tropical spastic paraparesis, *INF-α* interferon-alpha, *OMDS* Osame motor disability score, *SD* standard deviation

Regarding HAM/TSP treatment before screening, 32/33 (97.0%) and 25/32 (78.1%) patients received corticosteroids, and 7/33 (21.2%) and 8/32 (25.0%) patients received IFN-α in the mogamulizumab and placebo arm, respectively. In the mogamulizumab arm and placebo arm, 23/33 (69.7%) and 20/32 (62.5%) patients underwent maintenance corticosteroid therapy. The mean ± SD of OMDS was 4.7 ± 1.0 and 4.9 ± 0.9, and 20/33 (60.6%) and 22/32 (68.8%) patients had an OMDS of ≥ 5 in the mogamulizumab arm and the placebo arms, respectively.

The treatment compliance rate was 100% in both arms during the double-blind, open-label, and extension treatment periods. The mean ± SD cumulative mogamulizumab dose during the double-blind period was 0.57 ± 0.12 mg/kg. The mean ± SD overall duration of exposure to mogamulizumab until the final data cut-off was 105.09 ± 62.97 weeks (mogamulizumab arm) and 87.85 ± 57.23 weeks (placebo arm). The overall cumulative dose of mogamulizumab was 2.18 ± 1.53 mg/kg in the mogamulizumab arm through the double-blind, open-label, and extension treatment periods, and 1.70 ± 1.22 mg/kg in the placebo arm during the open-label and extension treatment periods. The mean ± SD dose of mogamulizumab per cycle was similar at 0.25 ± 0.08 mg/kg/cycle in the mogamulizumab arm and 0.24 ± 0.08 mg/kg/cycle in the placebo arm.

### Efficacy

#### Double-blind and open-label periods

The primary efficacy endpoint, the OMDS improvement rates were 15.2% (5/33 patients; mogamulizumab) and 18.8% (6/32 patients; placebo), with no significant difference (*p* = 0.708) (Table 2). All 11 patients in both arms had 1-grade improvements.

**Table 2:** Degree of improvement in OMDS in patients with HAM/TSP during the double-blind treatment period (full analysis set)

Degree of improvement in OMDS^a^	Mogamulizumab *n* = 33	Placebo *n* = 32	*p* value^b^
1	5 (15.2)	6 (18.8)	0.708
No improvement^c^	28 (84.8)	26 (81.3)	

Data are presented as *n* (%) of patients

^a^Lowest degree of improvement among the three time points in Cycle 2–Week 4, 8, and 12 in patients who showed improvement of ≥ 1 grade at all three time points

^b^Wilcoxon rank sum test using normal approximation

^c^Patients with no improvement at any time point in Cycle 2–Week 4, 8, or 12 and patients whose OMDS was missing at any point in Cycle 2–Week 4, 8, or 12

*HAM/TSP* human T-cell leukemia virus type 1-associated myelopathy/tropical spastic paraparesis, *OMDS* Osame motor disability score

**Fig. 3:**
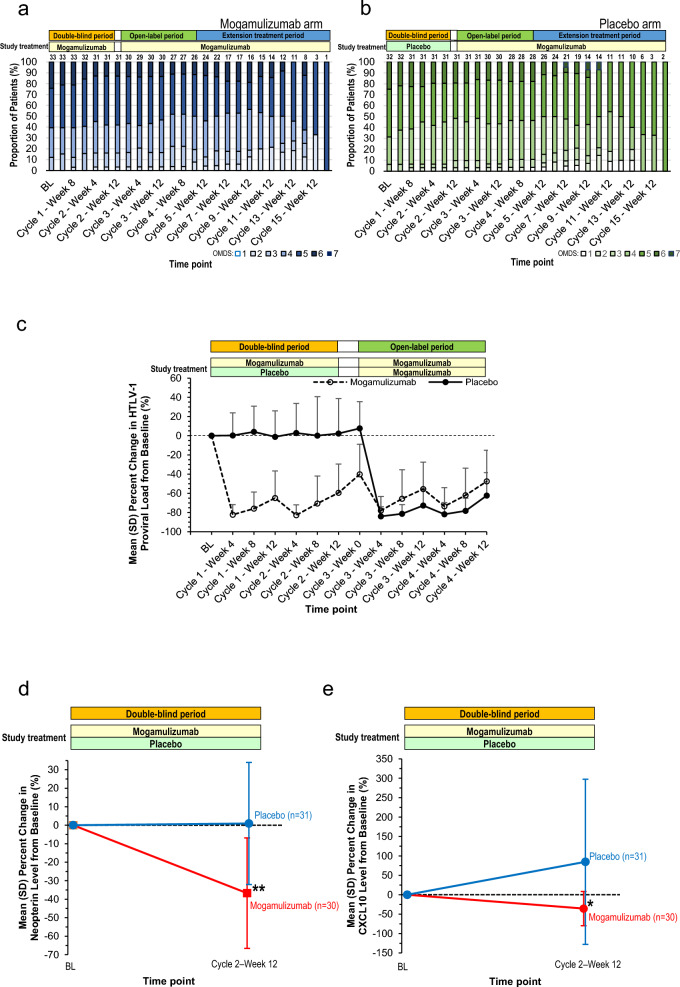
Trends in OMDS grade, peripheral HTLV-1 proviral load, and percent change in CSF neopterin and CXCL10 levels. **a**, **b** Trends in the proportion of patients by OMDS grade at each visit during the double-blind, open-label, and extension treatment periods. All patients in the placebo arm were treated with mogamulizumab in the open-label and extension treatment periods. The numbers at the top of each bar indicate the total number of patients included at each visit. **c** Trends in the mean ± SD percent change in the peripheral HTLV-1 proviral load at each visit from baseline during the double-blind and open-label periods. All patients in the placebo arm were treated with mogamulizumab in the open-label period. **d**, **e** Mean ± SD percent change in the CSF neopterin (**d**) and CXCL10 (**e**) levels at the end of the double-blind period (Cycle 2–Week 12) from baseline in patients treated with mogamulizumab or placebo. *p < 0.005; **p < 0.001. *BL* baseline, *OMDS* Osame motor disability score, *HTLV-1* human T-cell leukemia virus type 1, *SD* standard deviation, *CXCL10* chemokine C-X-C motif ligand 10

Regarding the main secondary endpoint, the mean ± SD improvement changes in the OMDS from baseline over Cycle 2–Weeks 4, 8, and 12 were 0.17 ± 0.37 (mogamulizumab) and 0.24 ± 0.41 (placebo), with no significant difference (− 0.06; 95% confidence interval, − 0.26 to 0.13; *p* = 0.517, *t*-test) (Table 3). The baseline OMDS grades were sustained and did not worsen through Cycle 4–Week 12 (Fig. 3a, b). No notable change in the 10-m walking time from baseline at Cycle 2–Week 12 was found (Supplementary Table 2).

**Table 3:** Mean OMDS in Cycle 2–Week 4, 8, and 12 and mean degree of improvement in Cycle 2–Week 4, 8, and 12 from baseline (full analysis set)

		Mogamulizumab *n* = 31	Placebo *n* = 31	*p* value
Mean OMDS	Mean ± SD	4.51 ± 0.99	4.63 ± 0.95	
Mean degree of improvement in OMDS from baseline	Mean ± SD	0.17 ± 0.37	0.24 ± 0.41	
	Difference (95% CI)		− 0.06 (− 0.26 to 0.13)	0.517

*CI* confidence interval, *OMDS* Osame motor disability score, *SD* standard deviation

The mean ± SD percentage change in the HTLV-1 proviral load in PBMCs (a pharmacological indication of the mogamulizumab efficacy) showed a substantial decrease (− 82.24 ± 10.50%) at Cycle 1–Week 4 from baseline. This decrease was sustained at a similar level (− 59.39 ± 29.91%) at Cycle 2–Week 12 in the mogamulizumab arm (Fig. 3c). In contrast, in the placebo arm, the mean ± SD percentage change in the HTLV-1 proviral load from baseline was 0.31 ± 23.43% and 2.32 ± 36.31% at Cycle 1–Week 4 and Cycle 2–Week 12, respectively, indicating that the HTLV-1 proviral load in the placebo arm remained at the baseline level during the double-blind period of mogamulizumab treatment. After dosing with mogamulizumab in the open-label treatment period, the HTLV-1 proviral load in the placebo arm decreased by approximately 60–80%, similar to the decrease observed in the mogamulizumab arm during the double-blind period.

The analyses of other secondary efficacy endpoints, namely MAS score, CGI-I score, overall improvement in VAS score, and evaluation of lower limb numbness/pain by VAS score, OABSS, and I-PSS showed no significant differences between the arms at Cycle 2–Week 12 (Supplementary Table 2).

The mean ± SD percent change in CSF neopterin level at Cycle 2–Week 12 from baseline was 0.92 ± 33.00% in the placebo arm, indicating almost no change, whereas that in the mogamulizumab arm decreased by − 36.73 ± 29.87%, which was a significant difference (*p* < 0.001) (Fig. 3d). The exploratory efficacy endpoint (mean ± SD change in the CSF CXCL10 level in the mogamulizumab arm at Cycle 2–Week 12 from baseline was − 35.62 ± 44.03% and significantly different vs the placebo arm (84.81 ± 212.63%) (*p* = 0.004) (Fig. 3e).

#### Extension treatment period

Throughout the extension treatment period after mogamulizumab dosing in all patients, no notable change was observed in the proportions of patients with OMDS improvement, no change, and worsening. Regarding the mean change in the OMDS at Cycle 8–Week 12 from baseline, a 1-grade improvement was observed in 5 (15.2%) patients, and no change was observed in 12 (36.4%) patients in the mogamulizumab arm. In contrast, a 2-grade improvement, 1-grade improvement, and no change was observed in 2 (6.3%), 6 (18.8%), and 11 (34.4%) patients, respectively, in the placebo arm (Fig. 3a, b). At Cycle 12–Week 12, a 1-grade improvement was observed in 5 (15.2%), no change was observed in 6 (18.2%), and a 1-grade worsening was observed in 1 (3.0%) patient in the mogamulizumab arm. A 2-grade improvement was observed in 2 (6.3%) patients, with a 1-grade improvement in 4 (12.5%) and no change in 5 (15.6%), in the placebo arm. No patients discontinued the study because of ≥ 2-grade OMDS worsening.

Regarding the HTLV-1 proviral load, the mean ± SD percent changes from baseline at Cycle 8 were − 59.73 ± 24.58% (*n* = 16) and − 65.48 ± 27.10% (*n* = 18) (mogamulizumab vs placebo arm, respectively) and those at Cycle 12 were − 54.94 ± 34.27% (*n* = 13) and − 67.67 ± 18.80% (*n* = 11), respectively. Therefore, the decrease in the HTLV-1 proviral load was sustained through the extension treatment period (Supplementary Fig. 1).

No significant worsening trend was found for the other efficacy endpoints through the extension treatment period (data not shown).

### Safety

During the double-blind period, all 34 (100%) patients in the mogamulizumab arm and 28/32 (87.5%) patients in the placebo arm experienced TEAEs. The most frequent TEAE was rash (17/34 (50.0%) patients) in the mogamulizumab arm and nasopharyngitis (6/32 (18.8%) patients) in the placebo arm. Overall, after mogamulizumab, 65/65 (100%) patients experienced TEAEs (Table 4); the most frequently observed TEAE (rash) occurred in 45/65 (69.2%) patients, followed by nasopharyngitis (25, 38.5%), decreased lymphocyte count (20, 30.8%), pyrexia (14, 21.5%), alopecia (13, 20.0%), back pain and stomatitis (12 patients each, 18.5%), arthralgia (11, 16.9%), and cystitis and contusion (10 patients each, 15.4%) (Table 5).

**Table 4:** Summary of the safety results (safety analysis set)

	Double-blind		Overall after dosing with mogamulizumab		
	Mogamulizumab arm	Placebo arm	Mogamulizumab arm	Placebo arm	Total
	*n* = 34	*n* = 32	*n* = 34	*n*^a^ = 31	*n*^a^ = 65
Patients with any TEAE	34 (100.0)	28 (87.5)	34 (100.0)	31 (100.0)	65 (100.0)
Death	1 (2.9)	0 (0.0)	1 (2.9)	0 (0.0)	1 (1.5)
Other serious TEAEs	3 (8.8)	3 (9.4)	16 (47.1)	10 (32.3)	26 (40.0)
Other significant TEAEs^b^	16 (47.1)	4 (12.5)	24 (70.6)	19 (61.3)	43 (66.2)

Data are presented as the *n* (%) of patients

^a^*n* was calculated based on the patients who received at least one dose of mogamulizumab. One patient in the placebo arm withdrew from the study because of a TEAE during the double-blind period

^b^TEAEs for which the action taken with mogamulizumab resulted in drug withdrawal, dose postponement, dose reduction, or dose interruption

*TEAE* treatment-emergent adverse event

**Table 5:** TEAEs that occurred in ≥ 3 patients in any treatment arm overall after dosing with mogamulizumab (safety analysis set)

	Overall after dosing with mogamulizumab					
	Mogamulizumab arm		Placebo arm		Total	
	Mogamulizumab *n* = 34		Mogamulizumab *n*^a^ = 31		Mogamulizumab *n*^a^ = 65	
	*n*	(%)	*n*	(%)	*n*	(%)
Patients with any TEAE	34	(100.0)	31	(100.0)	65	(100.0)
Blood and lymphatic system disorders	6	(17.6)	2	(6.5)	8	(12.3)
Lymphopenia	3	(8.8)	0	(0.0)	3	(4.6)
Eye disorders	12	(35.3)	9	(29.0)	21	(32.3)
Cataract	2	(5.9)	3	(9.7)	5	(7.7)
Gastrointestinal disorders	21	(61.8)	15	(48.4)	36	(55.4)
Stomatitis	7	(20.6)	5	(16.1)	12	(18.5)
Dental caries	3	(8.8)	3	(9.7)	6	(9.2)
Diarrhea	3	(8.8)	3	(9.7)	6	(9.2)
General disorders and administration site conditions	17	(50.0)	12	(38.7)	29	(44.6)
Pyrexia	9	(26.5)	5	(16.1)	14	(21.5)
Infections and infestations	25	(73.5)	26	(83.9)	51	(78.5)
Nasopharyngitis	13	(38.2)	12	(38.7)	25	(38.5)
Cystitis	5	(14.7)	5	(16.1)	10	(15.4)
Otitis externa	4	(11.8)	3	(9.7)	7	(10.8)
Tinea pedis	3	(8.8)	4	(12.9)	7	(10.8)
Urinary tract infection	4	(11.8)	1	(3.2)	5	(7.7)
Bronchitis	1	(2.9)	3	(9.7)	4	(6.2)
Gingivitis	3	(8.8)	1	(3.2)	4	(6.2)
Influenza	3	(8.8)	1	(3.2)	4	(6.2)
Tinea infection	3	(8.8)	1	(3.2)	4	(6.2)
Herpes zoster	0	(0.0)	3	(9.7)	3	(4.6)
Sinusitis	0	(0.0)	3	(9.7)	3	(4.6)
Injury, poisoning, and procedural complications	17	(50.0)	16	(51.6)	33	(50.8)
Contusion	8	(23.5)	2	(6.5)	10	(15.4)
Thermal burn	3	(8.8)	2	(6.5)	5	(7.7)
Investigations	20	(58.8)	15	(48.4)	35	(53.8)
Lymphocyte count decreased	11	(32.4)	9	(29.0)	20	(30.8)
Blood lactate dehydrogenase increased	4	(11.8)	2	(6.5)	6	(9.2)
White blood cell count decreased	4	(11.8)	1	(3.2)	5	(7.7)
Musculoskeletal and connective tissue disorders	20	(58.8)	12	(38.7)	32	(49.2)
Back pain	9	(26.5)	3	(9.7)	12	(18.5)
Arthralgia	8	(23.5)	3	(9.7)	11	(16.9)
Myalgia	3	(8.8)	1	(3.2)	4	(6.2)
Neoplasms: benign, malignant, and unspecified (including cysts and polyps)	5	(14.7)	2	(6.5)	7	(10.8)
Skin papilloma	3	(8.8)	1	(3.2)	4	(6.2)
Nervous system disorders	12	(35.3)	7	(22.6)	19	(29.2)
Headache	6	(17.6)	0	(0.0)	6	(9.2)
Skin and subcutaneous tissue disorders	31	(91.2)	30	(96.8)	61	(93.8)
Rash	20	(58.8)	25	(80.6)	45	(69.2)
Alopecia	6	(17.6)	7	(22.6)	13	(20.0)
Dermatitis contact	4	(11.8)	4	(12.9)	8	(12.3)
Eczema	4	(11.8)	4	(12.9)	8	(12.3)
Dry skin	6	(17.6)	1	(3.2)	7	(10.8)
Seborrheic dermatitis	2	(5.9)	5	(16.1)	7	(10.8)
Dermatitis	2	(5.9)	3	(9.7)	5	(7.7)
Pruritus	1	(2.9)	4	(12.9)	5	(7.7)
Drug eruption	3	(8.8)	0	(0.0)	3	(4.6)
Miliaria	0	(0.0)	3	(9.7)	3	(4.6)
Rash maculo-papular	3	(8.8)	0	(0.0)	3	(4.6)
Vascular disorders	5	(14.7)	1	(3.2)	6	(9.2)
Hypertension	3	(8.8)	0	(0.0)	3	(4.6)

^a^*n* was calculated based on the patients who received at least one dose of mogamulizumab. One patient in the placebo arm withdrew from the study because of a TEAE during the double-blind period

*TEAE* treatment-emergent adverse event

Skin and subcutaneous tissue disorders were the most common TEAEs (61, 93.8%), followed by infections and infestations (51, 78.5%); gastrointestinal disorders (36, 55.4%); investigations (35, 53.8%); injury, poisoning, and procedural complications (33, 50.8%); musculoskeletal and connective tissue disorders (32, 49.2%); and general disorders and administration site conditions (29, 44.6%).

During the double-blind period, grade ≥ 3 TEAEs occurred in 10/34 (29.4%) patients (mogamulizumab) [most frequent: decreased lymphocyte count: 5 (14.7%)] and 4/32 (12.5%) (placebo) (most frequent: decreased lymphocyte count, enterocolitis, hyponatremia, and transitional cell carcinoma, occurring in 1 (3.1%) patient each). Overall, after mogamulizumab, 32/65 (49.2%) patients experienced grade ≥ 3 TEAEs [most frequent: decreased lymphocyte count: 7 (10.8%)]. Compared with patients in the placebo arm, those in the mogamulizumab arm had a higher incidence of rash, alopecia, maculopapular rash, pyrexia, decreased lymphocyte count, decreased white blood cell count, and lymphopenia during the double-blind period.

In the mogamulizumab arm, a 77-year-old woman (1/34 [2.9%] patient) died of cardiac and cerebrovascular-related complications and aging on Day 212 during the double-blind period. A causal relationship of this event with mogamulizumab was denied by the investigator.

The first onsets of TEAEs were observed across the different treatment periods. Most TEAEs tended to occur for the first time in the early phase of each treatment period. No TEAEs showed an increased incidence during the extension treatment period. White blood cell and lymphocyte counts tended to decrease in the mogamulizumab arm, but other clinical investigations showed similar values between the arms. No clinically meaningful changes in the patients’ vital signs or electrocardiographic data were found throughout the study period.

## Discussion

In addition to the poor prognosis, the lack of effective treatments greatly impairs the quality of life in patients with HAM/TSP. Therefore, there is a dire need for an effective and clinically beneficial new treatment. With the aim of developing a better long-term treatment option for HAM/TSP, mogamulizumab was the first anti-CCR4 monoclonal antibody studied in affected patients.

The results of the double-blind period demonstrated no significant difference in the primary efficacy endpoint (OMDS improvement rate). Furthermore, no significant differences were found in the secondary efficacy endpoints or other clinical motor symptoms, namely the MAS score, overall improvement (CGI-I and VAS scores), bladder dysfunction (OABSS and I-PSS), and sensory dysfunction (VAS scores for lower extremity numbness and pain). While there was a clinical failure in the trial, there was a noted decrease in HTLV-1 proviral load and in CSF neopterin and CXCL10 levels.

Although it is difficult to compare the efficacy results between the previous phase 1/2a study and the present study owing to differences in the patients’ characteristics and study designs, as well as the small sample sizes, the previously demonstrated efficacy of mogamulizumab on motor activities was not verified after two intravenously administered doses of mogamulizumab at a 12-week interval in patients with HAM/TSP during the 24-week double-blind period.

Patients with HAM/TSP require unilateral support for walking at a median of approximately 8 years (OMDS of 5) after onset, require bilateral support at approximately 12.5 years, and become unable to walk at approximately 18 years [8]. HAM/TSP progresses slowly; historical Japanese patients’ data indicate that the OMDS deteriorates at an average of 0.2 grades per year [12]. Additionally, HAM/TSP symptoms progress slowly owing to spinal cord tissue damage caused by sustained inflammation and the poor regenerative ability of this tissue compared with other tissues. Therefore, it may take years for damaged nervous tissues to regenerate and recover motor functions. In this study, the primary endpoint was defined as the proportion of patients who showed a ≥ 1-grade improvement in the OMDS at all three time points in Cycle 2–Weeks 4, 8, and 12 from baseline. However, the 24-week evaluation period might not have been long enough to fully verify the treatment efficacy of mogamulizumab compared with placebo. Furthermore, mogamulizumab efficacy in suppressing disease progression as well as improvement, should have been evaluated. This is supported by the results of a 4-year long-term study showing that mogamulizumab had a long-term inhibitory effect on lower limb motor disability progression compared with a control cohort [20].

Similar to the trends in OMDS during the double-blind period, the proportions of patients with each OMDS grade showed no notable change after mogamulizumab during the open-label and the extension treatment periods, which had a mean treatment duration of 105 weeks (mogamulizumab) and 88 weeks (placebo). It is worth noting that considering that OMDS deteriorates at an average of 0.2 grades per year [12], the majority of our patients had 1- or 2-grade improvement or no change with approximately 2 years of mogamulizumab treatment. Therefore, mogamulizumab therapy might have prevented motor function from deteriorating further.

Although OMDS improvement was insufficient, significant decreases were found in the HTLV-1 proviral load in PBMCs, CSF neopterin level, and CSF CXCL10 level with mogamulizumab vs. placebo. The HTLV-1 proviral load in PBMCs decreased rapidly by ≥ 80% 4 weeks after the first mogamulizumab administration, and a 60–80%-reduction was sustained by repeat mogamulizumab administration at 12-week intervals throughout the open-label and extension treatment periods. These results are similar to previous results [19, 20] that showed similar trends of reduction up to 85 days after a single administration of mogamulizumab and after repeat administration, with a trough 7–29 days after administration (because of the time resolution, this occurred 4 weeks after administration in the present study). The decreased CSF neopterin and CXCL10 levels in this study are also consistent with previous results [19, 20]. Therefore, our results confirmed and further extended the efficacy of mogamulizumab in decreasing HTLV-1 proviral load and the CSF levels of the two inflammatory biomarkers. It has been suggested that the HTLV-1 proviral load in PBMCs and the CSF neopterin or CXCL10 level correlate with HAM/TSP disease activity [4, 11, 22–25]. The current results showed that mogamulizumab decreased the HTLV-1 proviral load and maintained low inflammatory biomarkers levels, thereby suppressing progressive inflammation. Therefore, it is anticipated that lowering and maintaining low CSF neopterin and CXCL10 levels will lead to improvement and maintenance of the long-term prognosis as demonstrated in corticosteroid-treated patients with HAM/TSP [26–28]. This also suggests that initiating mogamulizumab treatment early, before severe symptoms, might improve outcomes in patients with HTLV-1 infection. Although direct comparisons are challenging because of differing trial designs, the effectiveness of steroids in treating HAM/TSP has been previously suggested and evidenced in several studies [2, 29, 30]. Steroids are commonly used for their direct anti-inflammatory properties. In contrast, mogamulizumab works by reducing the number of infected cells, thereby indirectly suppressing inflammation. This mechanism implies that mogamulizumab’s therapeutic effects might manifest over a longer period compared with steroids. Additionally, our study showed that mogamulizumab can further decrease inflammatory markers in the CSF in patients who are already receiving steroid therapy, suggesting the potential for an additive or synergistic effect in combination with steroids. This finding is particularly important, given that some HAM/TSP patients do not achieve adequate control of spinal inflammation with steroid therapy alone [27]. Additionally, in some cases, long-term steroid therapy does not necessarily translate into improved long-term outcomes [12]. Given these observations, it is clear that mogamulizumab could play a crucial role in the management of HAM/TSP, especially in cases where steroids alone are insufficient. This aligns with the growing demand for new therapeutic options that offer additional benefits beyond existing treatments. However, we acknowledge that further research, including comparative studies, is necessary to fully understand the benefits and limitations of mogamulizumab in the context of HAM/TSP treatment.

The analysis of the present safety results identified a ≥ 10% higher incidence of TEAEs with mogamulizumab vs. placebo during the double-blind period. Additionally, decreased white blood cell count, lymphopenia, alopecia, and maculopapular rash developed in ≥ 3 (8.8%) patients in the mogamulizumab arm but in none in the placebo arm. Overall, after mogamulizumab, the major TEAEs of rash, nasopharyngitis, and decreased lymphocyte count were reported in 30–70% of patients. However, most of these TEAEs were grade ≤ 3, and the safety profile was considered clinically acceptable in most cases. In summary, the present safety profile was similar to that reported in the previous phase 1/2a study [19]. However, the incidence rates of some typical TEAEs, including rash, in this study were higher than those in the previous phase 1/2a study. The higher incidences in this study can likely be attributed to the longer study period, the dosing criteria for resuming the study treatment, and the higher initial mogamulizumab dose of 0.3 mg/kg. In the previous study, the mogamulizumab dose was 0.03 mg/kg, which is considered the minimum effective dose in long-term treatment, considering safety. Because HAM/TSP is a chronic disease, future studies should investigate the long-term efficacy and safety of 0.03 mg/kg mogamulizumab administered at ≥ 3-month intervals.

In this trial, the sample size was determined on the basis of the efficacy observed in the phase 1/2a trial of mogamulizumab for HAM/TSP, as well as the improvement rate in the placebo group from the IFN-α trial for HAM/TSP. Notably, a significant placebo effect observed in our trial suggests the necessity of developing outcome measures less susceptible to such effects. HAM/TSP, which is a rare disease with a limited patient population and characterized by irreversible neurological damage, presents substantial challenges in therapeutic development. The optimal design for clinical trials targeting “symptom improvement” as a primary endpoint would involve early-stage patients (within 5 years of onset) who have high disease activity but have not yet developed complete neurological damage. However, even in Japan, with its relatively large patient population, recruiting suitable candidates for a rare disease, such as HAM/TSP, is difficult. An international collaborative trial was considered, but was deemed unfeasible owing to the very low number of patients with HAM/TSP in collaborating countries and the uncertainty of performing such a trial with the necessary effort and budget. Furthermore, trials focusing on “symptom progression inhibition” require a long-term approach, potentially over 5 years, to observe significant changes in OMDS in patients on continuous steroid therapy. Ethical concerns and budget constraints related to maintaining a placebo group for such a duration led to a decision against this trial design. The development of treatment options for HAM/TSP may necessitate more flexible regulatory approaches. This could include the acceptance of alternative endpoints, such as biomarkers, as primary measures, allowing for conditional approval based on efficacy and safety demonstrated in small patient groups over short study durations. Comprehensive validation of clinical effectiveness could then be achieved through real-world data analysis.

In conclusion, the present results demonstrated a small improvement in clinical symptoms with mogamulizumab compared with placebo during the 24 weeks of double-blind, open-label, and extension treatment periods. This study has limitations, namely the small sample size and relatively short 24-week double-blind treatment period. These limitations might have prevented full evaluation of the clinical efficacy of mogamulizumab on motor activities in patients with HAM/TSP. However, this study showed significant efficacy of mogamulizumab in decreasing the HTLV-1 proviral load and CSF neopterin and CXCL10 levels without new safety concerns in patients with an OMDS of 3–6. Considering the clinical implications of significantly reduced HTLV-1 proviral load and CSF neopterin and CXCL10 levels in this study, a future study using real-world data as a control is expected to investigate the long-term efficacy of mogamulizumab in suppressing HAM/TSP disease progression.

### Supplementary Information

Below is the link to the electronic supplementary material.

Supplementary file1 (DOCX 266 KB)

## Data Availability

The corresponding author has full access to all data, and anonymized data will be shared by reasonable request from any qualified investigator.

## References

[1] Gessain A, Cassar O (2012). Epidemiological aspects and world distribution of HTLV-1 infection. Front Microbiol.

[2] Bangham CRM, Araujo A, Yamano Y (2015). HTLV-1-associated myelopathy/tropical spastic paraparesis. Nat Rev Dis Primers.

[3] Osame M, Usuku K, Izumo S (1986). HTLV-I associated myelopathy, a new clinical entity. Lancet.

[4] Nakagawa M, Izumo S, Ijichi S (1995). HTLV-I-associated myelopathy: analysis of 213 patients based on clinical features and laboratory findings. J Neurovirol.

[5] Olindo S, Cabre P, Lézin A (2006). Natural history of human T-lymphotropic virus 1-associated myelopathy: a 14-year follow-up study. Arch Neurol.

[6] Martin F, Fedina A, Youshya S (2010). A 15-year prospective longitudinal study of disease progression in patients with HTLV-1 associated myelopathy in the UK. J Neurol Neurosurg Psychiatry.

[7] Yamano Y, Sato T (2012). Clinical pathophysiology of human T-lymphotropic virus-type 1-associated myelopathy/tropical spastic paraparesis. Front Microbiol.

[8] Coler-Reilly ALG, Yagishita N, Suzuki H (2016). Nation-wide epidemiological study of Japanese patients with rare viral myelopathy using novel registration system (HAM-net). Orphanet J Rare Dis.

[9] Satake M, Yamaguchi K, Tadokoro K (2012). Current prevalence of HTLV-1 in Japan as determined by screening of blood donors. J Med Virol.

[10] Kaplan JE, Osame M, Kubota H (1990). (1990) The risk of development of HTLV-I-associated myelopathy/tropical spastic paraparesis among persons infected with HTLV-I. J Acquir Immune Defic Syndr (1988).

[11] Sato T, Yagishita N, Tamaki K (2018). Proposal of classification criteria for HTLV-1-associated myelopathy/tropical spastic paraparesis disease activity. Front Microbiol.

[12] Tsutsumi S, Sato T, Yagishita N (2019). Real-world clinical course of HTLV-1-associated myelopathy/tropical spastic paraparesis (HAM/TSP) in Japan. Orphanet J Rare Dis.

[13] Ishida T, Joh T, Uike N (2012). Defucosylated anti-CCR4 monoclonal antibody (KW-0761) for relapsed adult T-cell leukemia-lymphoma: a multicenter phase II study. J Clin Oncol.

[14] Kasamon YL, Chen H, de Claro RA (2019). FDA approval summary: mogamulizumab-kpkc for mycosis fungoides and Sézary syndrome. Clin Cancer Res.

[15] European Commission (2018) Summary of European Union decisions on marketing authorisations in respect of medicinal products from 1 November 2018 to 30 November 2018 (Published pursuant to Article 13 or Article 38 of Regulation (EC) No 726/2004 of the European Parliament and of the Council). Off J Eur Union C 465:01. http://eur-lex.europa.eu/legal-content/EN/TXT/PDF/?uri=OJ:C:2018:465:FULL&from=EN. Accessed 8 June 2022

[16] Hieshima K, Nagakubo D, Nakayama T (2008). Tax-inducible production of CC chemokine ligand 22 by human T cell leukemia virus type 1 (HTLV-1)-infected T cells promotes preferential transmission of HTLV-1 to CCR4-expressing CD4+ T cells. J Immunol.

[17] Yamano Y, Araya N, Sato T (2009). Abnormally high levels of virus-infected IFN-gamma+ CCR4+ CD4+ CD25+ T cells in a retrovirus-associated neuroinflammatory disorder. PLoS One.

[18] Araya N, Sato T, Ando H (2014). HTLV-1 induces a Th1-like state in CD4+CCR4+ T cells. J Clin Investig.

[19] Sato T, Coler-Reilly ALG, Yagishita N (2018). Mogamulizumab (anti-CCR4) in HTLV-1-associated myelopathy. N Engl J Med.

[20] Sato T, Yamauchi J, Yagishita N (2023). Long-term safety and efficacy of mogamulizumab (anti-CCR4) for treating virus-associated myelopathy. Brain.

[21] Izumo S, Goto I, Itoyama Y (1996). Interferon-alpha is effective in HTLV-I-associated myelopathy: a multicenter, randomized, double-blind, controlled trial. Neurology.

[22] Nomoto M, Utatsu Y, Soejima Y (1991). Neopterin in cerebrospinal fluid: a useful marker for diagnosis of HTLV-I-associated myelopathy/tropical spastic paraparesis. Neurology.

[23] Nagai M, Usuku K, Matsumoto W (1998). Analysis of HTLV-I proviral load in 202 HAM/TSP patients and 243 asymptomatic HTLV-I carriers: high proviral load strongly predisposes to HAM/TSP. J Neurovirol.

[24] Ando H, Sato T, Tomaru U (2013). Positive feedback loop via astrocytes causes chronic inflammation in virus-associated myelopathy. Brain.

[25] Sato T, Coler-Reilly A, Utsunomiya A (2013). CSF CXCL10, CXCL9, and neopterin as candidate prognostic biomarkers for HTLV-1-associated myelopathy/tropical spastic paraparesis. PLoS Negl Trop Dis.

[26] Tamaki K, Sato T, Tsugawa J (2019). Cerebrospinal fluid CXCL10 as a candidate surrogate marker for HTLV-1-associated myelopathy/tropical spastic paraparesis. Front Microbiol.

[27] Yamauchi J, Sato T, Yagishita N (2020). Use of cerebrospinal fluid CXCL10 and neopterin as biomarkers in HTLV-1-associated myelopathy/tropical spastic paraparesis treated with steroids. J Neurol Neurosurg Psychiatry.

[28] Yamauchi J, Araya N, Yagishita N (2021). An update on human T-cell leukemia virus type I (HTLV-1)-associated myelopathy/tropical spastic paraparesis (HAM/TSP) focusing on clinical and laboratory biomarkers. Pharmacol Ther.

[29] Croda MG, de Oliveira AC, Vergara MP, Bonasser F, Smid J, Duarte AJ, Casseb J (2008). Corticosteroid therapy in TSP/HAM patients: the results from a 10 years open cohort. J Neurol Sci.

[30] Coler-Reilly ALG, Sato T, Matsuzaki T (2017). Effectiveness of daily prednisolone to slow progression of human T-lymphotropic virus type 1-associated myelopathy/tropical spastic paraparesis: a multicenter retrospective cohort study. Neurotherapeutics.

