# Supplementary material for: Multicenter, randomized, double-blind, placebo-controlled phase 3 study of mogamulizumab with open-label extension study in a minimum number of patients with human T-cell leukemia virus type-1-associated myelopathy
Source: J Neurol. 2024 Mar 2;271(6):3471–85. doi: 10.1007/s00415-024-12239-x (PMC11136778; doi:10.1007/s00415-024-12239-x)
Supplement: Supplementary file 1 — Supplementary file1 (DOCX 266 KB) [file 415_2024_12239_MOESM1_ESM.docx]

**Multicenter, randomized, double-blind, placebo-controlled phase 3 study of mogamulizumab with open-label extension study in a minimum number of patients with Human T-cell leukemia virus type-1-associated myelopathy**

*Journal of Neurology*

Tomoo Sato^1,2^, Masahiro Nagai^3^, Osamu Watanabe^4^, Tatsuro Misu^5^, Norihiro Takenouchi^6^, Ryuichi Ohkubo^7^, Satoshi Ishihara^8^, Yoshio Tsuboi^9^, Masahisa Katsuno^10^, Masanori Nakagawa^11^, Takuya Matsushita^12^, Yasuhiro Aso^13^, Eiji Matsuura^14^, Takashi Tokashiki^15^, Akihiro Mukaino^16^, Hiroaki Adachi^17^, Kaoru Nakanishi^18^, Yusuke Yamaguchi^18^, Saaya Yamaguchi^18^, Yoshihisa Yamano^1,2^

**Corresponding author:**

Yoshihisa Yamano

Department of Rare Diseases Research, Institute of Medical Science, St. Marianna University School of Medicine, Kawasaki, Japan

Department of Neurology, St. Marianna University School of Medicine, Kawasaki, Japan

E-mail address: yyamano@marianna-u.ac.jp

**This file contains the details of the Osame motor disability score.**

**Supplementary Table 1** Osame motor disability score

| **Grade** | **Motor disability** |
| --- | --- |
| 0 | No walking or running abnormalities |
| 1 | Normal gait but runs slowly |
| 2 | Abnormal gait (stumbling, stiffness) |
| 3 | Unable to run |
| 4 | Needs handrail to climb stairs |
| 5 | Needs a cane (unilateral support) to walk |
| 6 | Needs bilateral support to walk |
| 7 | Can walk 5-10 m with bilateral support |
| 8 | Can walk 1-5 m with bilateral support |
| 9 | Cannot walk, but able to crawl |
| 10 | Cannot crawl, but able to move using arms |
| 11 | Cannot move around, but able to turn over in bed |
| 12 | Cannot turn over in bed |
| 13 | Cannot even move toes |

**Supplementary Table 2** Results of the secondary efficacy endpoints at Cycle 2-Week 12 (full analysis set)

|  |  |  | Actual | | Change from baseline | | p value |
| --- | --- | --- | --- | --- | --- | --- | --- |
|  |  |  | Mogamulizumab n = 33 | Placebo n = 32 | Mogamulizumab n = 33 | Placebo n = 32 |  |
| 10-m timed walk test, seconds | n |  | 31 | 31 | 31 | 31 |  |
|  | Mean ± SD |  | 18.95 ± 16.26 | 14.82 ± 9.20 | −0.88 ± 5.57 | −1.18 ± 4.19 |  |
|  | Difference (95% CI) |  | – | – | – | 0.29 (−2.21 to 2.80) | 0.815 |
| MAS, over Cycle 2-Week 4, 8, 12 | n |  | – | – | 31 | 31 |  |
|  | Mean ± SD |  | – | – | −0.32 ± 0.69 | −0.52 ± 0.84 |  |
|  | Difference (95% CI) |  | – | – | – | −0.20 (−0.59 to 0.19) | 0.317 |
| CGI-I score | n |  | 31 | 31 | – | – |  |
|  | Mean ± SD |  | 3.6 ± 0.8 | 3.6 ± 0.8 | – | – |  |
| VAS score for HAM/TSP, mm | n |  | 31 | 31 | 31 | 31 |  |
|  | Mean ± SD |  | 57.2 ± 28.5 | 60.5 ± 29.3 | −7.3 ± 22.9 | −2.5 ± 21.9 |  |
|  | Difference (95% CI) |  |  |  |  | −4.8 (−16.2 to 6.5) | 0.398 |
| OABSS | n |  | 23 | 24 | 23 | 24 |  |
|  | Mean ± SD |  | 6.0 ± 3.6 | 5.8 ± 3.5 | −0.3 ± 2.3 | −0.4 ± 2.0 |  |
|  | Difference (95% CI) |  | – | – | – | 0.1 (−1.1 to 1.4) | 0.855 |
| I-PSS | n |  | 23 | 24 | 23 | 24 |  |
|  | Mean ± SD |  | 12.7 ± 8.7 | 14.0 ± 9.3 | −2.7 ± 7.0 | 0.3 ± 5.6 |  |
|  | Difference (95% CI) |  | – | – | – | −3.0 (−6.7 to 0.7) | 0.110 |
| Lower extremity numbness (VAS score), mm | n |  | 31 | 31 | 31 | 31 |  |
|  | Mean ± SD |  | 37.3 ± 35.2 | 39.4 ± 35.2 | −2.1 ± 20.2 | 0.3 ± 18.0 |  |
|  | Difference (95% CI) |  | – | – | – | −2.4 (−12.1 to 7.4) | 0.629 |
| Lower extremity pain (VAS score), mm | n |  | 31 | 31 | 31 | 31 |  |
|  | Mean ± SD |  | 29.9 ± 34.6 | 30.3 ± 30.9 | 1.2 ± 24.6 | 1.5 ± 17.1 |  |
|  | Difference (95% CI) |  | – | – | – | −0.3 (−11.0 to 10.5) | 0.961 |

*CGI-I* Clinical Global Impression-Improvement Scale, *CI* confidence interval, *HAM/TSP* human T-cell leukemia virus type 1-associated myelopathy/tropical spastic paraparesis, *I-PSS* International Prostate Symptom Score, *MAS* modified Ashworth scale, *OABSS* Overactive Bladder Symptom Score, *SD* standard deviation, *VAS* visual analogue scale

**Supplementary Figure 1** Trends in the percent change in the peripheral HTLV-1 proviral load at each visit from baseline during the double-blind, open-label, and extension treatment periods


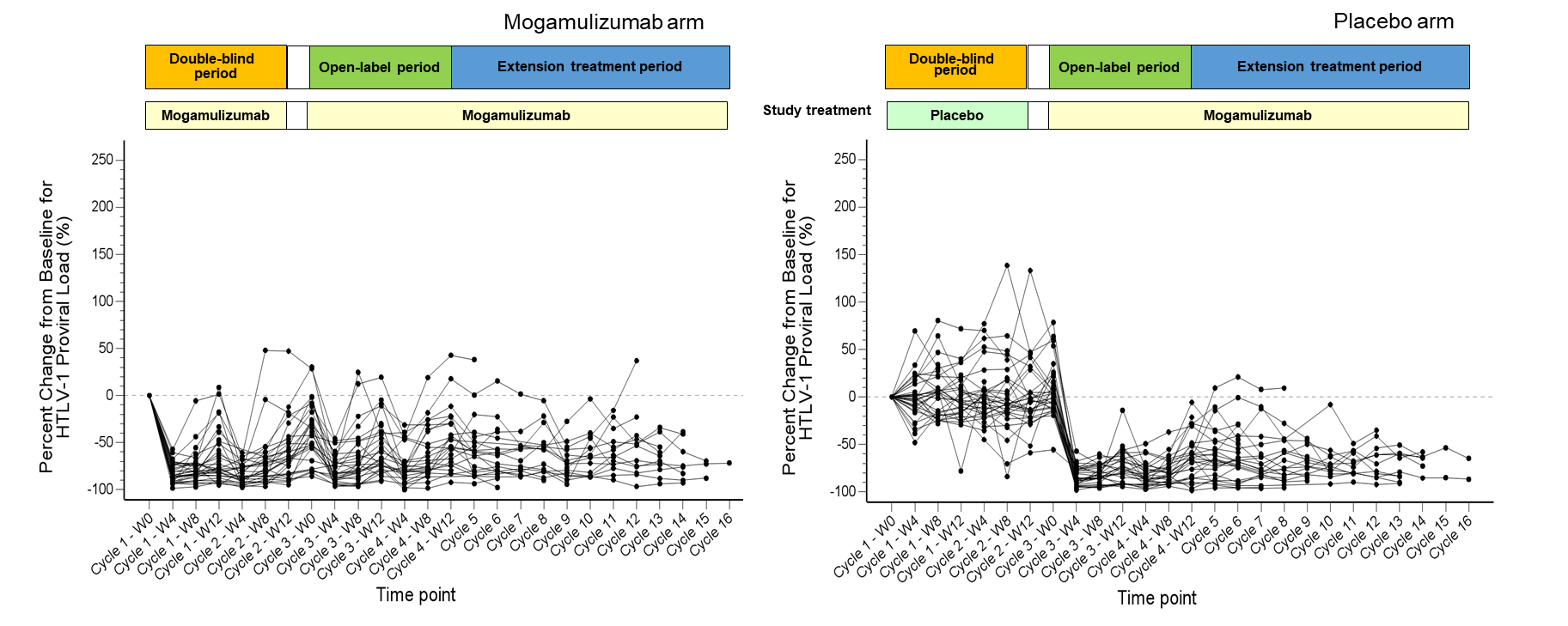


All patients in the placebo arm were treated with mogamulizumab in the open-label and extension treatment periods. *HTLV-1* human T-cell leukemia virus type 1
